# Supplementary material for: SavvyCNV: Genome-wide CNV calling from off-target reads
Source: PLoS Comput Biol. 2022 Mar 16;18(3):e1009940. doi: 10.1371/journal.pcbi.1009940 (PMC8959187; doi:10.1371/journal.pcbi.1009940)
Supplement: S1 Text — Table A. Benchmarking off-target CNV calling from targeted panel data. The table shows the performance of the different CNV calling software based on the size of the CNV. The tools were run with multiple different parameters. For this comparison, we have selected the configuration for each tool that provides the highest recall with a precision of at least 50%. More variants may be detected by each tool with different configuration, but with precision less than 50%. Table B. Benchmarking on-target CNV calling from the ICR96 targeted panel data. The table shows the performance of the different CNV calling software based on the size of the CNV. The tools were run with multiple different parameters. For this comparison, we have selected the configuration for each tool that provides the highest recall with a precision of at least 50%. Table C. Benchmarking on-target CNV calling from the exome data. The table shows the performance of the different CNV calling software based on the size of the CNV. The tools were run with multiple different parameters. For this comparison, we have selected the configuration for each tool that provides the highest recall with a precision of at least 50%. Fig A. An example of a single large CNV called by SavvyCNV in chromosome 8. The CNV is a heterozygous deletion of 8:6,800,000–12,400,000, which is a 5.6Mbp deletion. The CNV analysis uses a bin size of 200kbp, so the CNV covers 28 bins. The normalised read depth of each bin is shown as an error bar, where the error is the estimated error calculated by SavvyCNV. Bins either side of the deletion have a normal normalised read depth near 1, whereas bins inside the deletion have a normalised read depth near 0.5. This CNV overlaps a targeted gene in the sequencing capture, and this targeted data covers two separate bins, which are marked. Note that the error estimate for these two bins is slightly smaller than the other bins, partly because of the increased read count contributed by the targeted [file pcbi.1009940.s001.docx]

# SavvyCNV: genome-wide CNV calling from off-target reads

# Supplementary information

Table A. Benchmarking off-target CNV calling from targeted panel data.

The table shows the performance of the different CNV calling software based on the size of the CNV. The tools were run with multiple different parameters. For this comparison, we have selected the configuration for each tool that provides the highest recall with a precision of at least 50%. More variants may be detected by each tool with different configuration, but with precision less than 50%.

| **Size** | **CNVs** | **Software** | **True positives** | **False positives** | **Recall** | **Precision** | **Caller options used** | **Optimised filters used** |
| --- | --- | --- | --- | --- | --- | --- | --- | --- |
| All sizes | 267 | SavvyCNV | 68 | 67 | 25.5% | 50.4% | Bin size: 200kbp, transition probability: 0.001, min reads: 25 | Quality >= 88.36, quality per bin >= 19.53, bin count >= 3 |
|  |  | GATK gCNV | 40 | 38 | 15% | 51.3% | Bin size: 300kbp | Quality >= 25.74, quality per bin >= 4.867, bin count >= 3 |
|  |  | DeCON | 41 | 40 | 15.4% | 50.6% | Bin size: 200kbp, transition probability: 0.01, min reads: 40 | Bin count >= 3, BF >= 7.85, BF per CNV size >= 1.067×10^-5^ |
|  |  | Excavator2 | 30 | 20 | 11.2% | 60.0% | Bin size: 150kbp | ProbCall >= 0.5142, CNV size >= 3.6Mbp |
|  |  | CnvKit | 27 | 23 | 10.1% | 54.0% | Bin size: 400kbp | Bin count >= 21, weight >= 15.52, weight per CNV size >= 1.067×10^-6^ |
|  |  | CopywriteR | 30 | 4 | 11.2% | 88.2% | Bin size: 200kbp | Bin count >= 2, abs(log(dosage)) >= 0.4128, abs(log(dosage))*bin count >= 12.98 |
| >=1Mb (including >=5Mb) | 42 | SavvyCNV | 41 | 11 | 97.6% | 78.8% | Bin size: 200kbp, transition probability: 0.01, min reads: 45 | Quality >= 85.76, quality per bin >= 20.99, bin count >= 5 |
|  |  | GATK gCNV | 36 | 28 | 85.7% | 56.3% | Bin size: 300kbp | Quality >= 51.08, quality per bin >= 3.967, bin count >= 3 |
|  |  | DeCON | 36 | 31 | 85.7% | 53.7% | Bin size: 300kbp, transition probability: 0.1, min reads: 35 | Bin count >= 2, BF > 15.4, BF per CNV size >= 6.593×10^-6^ |
|  |  | Excavator2 | 29 | 19 | 69.0% | 60.4% | Bin size: 150kbp | ProbCall >= 0.6207, CNV size >= 3.6Mbp |
|  |  | CnvKit | 26 | 24 | 61.9% | 52.0% | Bin size: 400kbp | Bin count >= 21, weight > 15.52, weight per CNV size >= 1.067×10^-6^ |
|  |  | CopywriteR | 30 | 26 | 71.4% | 53.6% | Bin size: 200kbp | Bin count >= 12, abs(log(dosage)) >= 0.4128, abs(log(dosage))*bin count >= 9.074 |
| >=5Mb | 26 | SavvyCNV | 26 | 0 | 100% | 100% | Bin size: 300kbp, transition probability: 0.01, min reads: 40 | Quality >= 81.18, quality per bin >= 18.36, bin count >= 18 |
|  |  | GATK gCNV | 24 | 8 | 92.3% | 75% | Bin size: 300kbp | Quality per bin >= 3.586, bin count >= 14 |
|  |  | DeCON | 26 | 19 | 100% | 74.3% | Bin size: 200kbp, transition probability: 0.1, min reads: 40 | Bin count >= 5, BF >= 8.37, BF per CNV size >= 7.775×10^-6^ |
|  |  | Excavator2 | 26 | 3 | 100% | 89.7% | Bin size: 200kbp | ProbCall >= 0.8170, CNV size >= 5.6Mbp |
|  |  | CnvKit | 25 | 23 | 96.2% | 52.1% | Bin size: 250kbp | Bin count >= 32, weight >= 20.47, weight per CNV size >= 1.443×10^-6^ |
|  |  | CopywriteR | 26 | 2 | 100% | 92.9% | Bin size: 100kbp | Bin count >= 41, abs(log(dosage)) >= 0.4481, abs(log(dosage))*bin count >= 27.71 |

Table B Benchmarking on-target CNV calling from the ICR96 targeted panel data.

The table shows the performance of the different CNV calling software based on the size of the CNV. The tools were run with multiple different parameters. For this comparison, we have selected the configuration for each tool that provides the highest recall with a precision of at least 50%.

| **Size** | **CNVs** | **Software** | **True positives** | **False positives** | **Recall** | **Precision** | **Caller options used** | **Optimised filters used** |
| --- | --- | --- | --- | --- | --- | --- | --- | --- |
| All (single exon and multi-exon) | 68 | SavvyCNV | 67 | 42 | 98.5% | 61.5% | Bin size: 200bp, transition probability 0.1, min reads: 30 | Quality >= 31.53, quality per CNV size >= 0.00386, bin count >= 2 |
|  |  | GATK gCNV | 66 | 11 | 97.1% | 85.7% | Bins are exons | Quality >= 2.932, quality per bin >= 1.466 |
|  |  | DeCON | 65 | 65 | 95.6% | 50.0% | Bins are exons, transition probability: 0.3 | BF >= 2.0, BF per CNV size >= 6.459×10^-6^ |
|  |  | CnvKit | 12 | 12 | 17.6% | 50.0% | Bins are exons | Bin count >= 17, weight >= 6.912, weight per CNV size >= 1.052×10^-6^ |
| Multi-exon | 43 | SavvyCNV | 43 | 15 | 100% | 74.1% | Bin size: 400bp, transition probability 0.1, min reads: 40 | Quality >= 36.30, quality per bin >= 18.15, bin count >= 2 |
|  |  | GATK gCNV | 43 | 11 | 100% | 79.6% | Bins are exons | Quality >= 2.932, quality per bin >= 1.466 |
|  |  | DeCON | 42 | 14 | 97.7% | 75.0% | Bins are exons, transition probability: 0.1 | Bin count >= 2, BF >= 2.0, BF per CNV size >= 6.459×10^-5^ |
|  |  | CnvKit | 10 | 8 | 23.2% | 55.5% | Bins are exons | Bin count >= 25, weight >= 8.330, weight per CNV size >= 1.052×10^-6^ |
| Without detectable breakpoint | 61 | SavvyCNV | 61 | 42 | 100% | 59.2% | Bin size: 200bp, transition probability: 0.1, min reads: 30 | Quality >= 31.53, quality per CNV size >= 0.00386, bin count >= 2 |
|  |  | GATK gCNV | 60 | 11 | 98.4% | 84.5% | Bins are exons | Quality >= 2.932, quality per bin >= 1.466 |
|  |  | DeCON | 58 | 45 | 95.1% | 56.3% | Bins are exons, transition probability 0.01 | BF >= 2.91, BF per CNV size >= 6.459×10^-5^ |
|  |  | CnvKit | 10 | 8 | 16.4% | 55.5% | Bins are exons | Bin count >= 25, weight >= 8.330, weight per CNV size >= 1.052×10^-6^ |

Table C. Benchmarking on-target CNV calling from the exome data.

The table shows the performance of the different CNV calling software based on the size of the CNV. The tools were run with multiple different parameters. For this comparison, we have selected the configuration for each tool that provides the highest recall with a precision of at least 50%.

| **Size** | **CNVs** | **Software** | **True positives** | **False positives** | **Recall** | **Precision** | **Caller options used** | **Optimised filters used** |
| --- | --- | --- | --- | --- | --- | --- | --- | --- |
| All sizes | 75 | SavvyCNV | 65 | 58 | 86.7% | 52.8% | Bin size: 8kbp, transition probability: 0.03, min reads: 30 | Quality >= 104.5, quality per bin >= 4.964, bin count >= 12 |
|  |  | GATK gCNV | 6 | 5 | 8.0% | 54.5% | Bin size: 12kbp | Quality >= 36.86, quality per bin >= 18.58, bin count >= 20 |
|  |  | DeCON | 35 | 34 | 46.7% | 50.7% | Bin size: 15kbp, transition probability: 0.1, min reads: 45 | Bin count >= 11, BF >= 20.1, BF per CNV size >= 8.302×10^-5^ |
|  |  | Excavator2 | 6 | 6 | 8.0% | 50.0% | Bin size: 25kbp | ProbCall >= 0.9951, CNV size >= 250kbp |
|  |  | CnvKit | 3 | 1 | 4.0% | 75.0% | Bin size: 25kbp | Weight >= 85.54, weight per CNV size >= 8.423×10^-5^ |
|  |  | CopywriteR | 4 | 4 | 5.3% | 50% | Bin size: 20kbp | Bin count >= 19, abs(log(dosage)) > 0.9713, abs(log(dosage))*bin count >= 21.93 |
| >=200kb | 40 | SavvyCNV | 35 | 34 | 87.5% | 50.7% | Bin size: 40kbp, transition probability: 0.03, min reads: 50 | Quality >= 40.31, quality per bin >= 5.798, bin count >= 5 |
|  |  | GATK gCNV | 6 | 5 | 15.0% | 54.5% | Bin size: 40kbp | Quality >= 671.9, quality per bin >= 39.52, bin count >= 8 |
|  |  | DeCON | 25 | 24 | 62.5% | 51.0% | Bin size: 12kbp, transition probability: 0.01, min reads: 20 | Bin count >= 19, BF >= 22.5, BF per CNV size >= 8.995×10^-5^ |
|  |  | Excavator2 | 6 | 6 | 15.0% | 50.0% | Bin size: 25kbp | ProbCall >= 0.9951, CNV size >= 250kbp |
|  |  | CnvKit | 3 | 1 | 7.5% | 75.0% | Bin size: 25kbp | Weight >= 85.54, weight per CNV size >= 8.423×10^-5^ |
|  |  | CopywriteR | 4 | 4 | 10% | 50% | Bin size: 20kbp | Bin count >= 19, abs(log(dosage)) > 0.9713, abs(log(dosage))*bin count >= 21.93 |


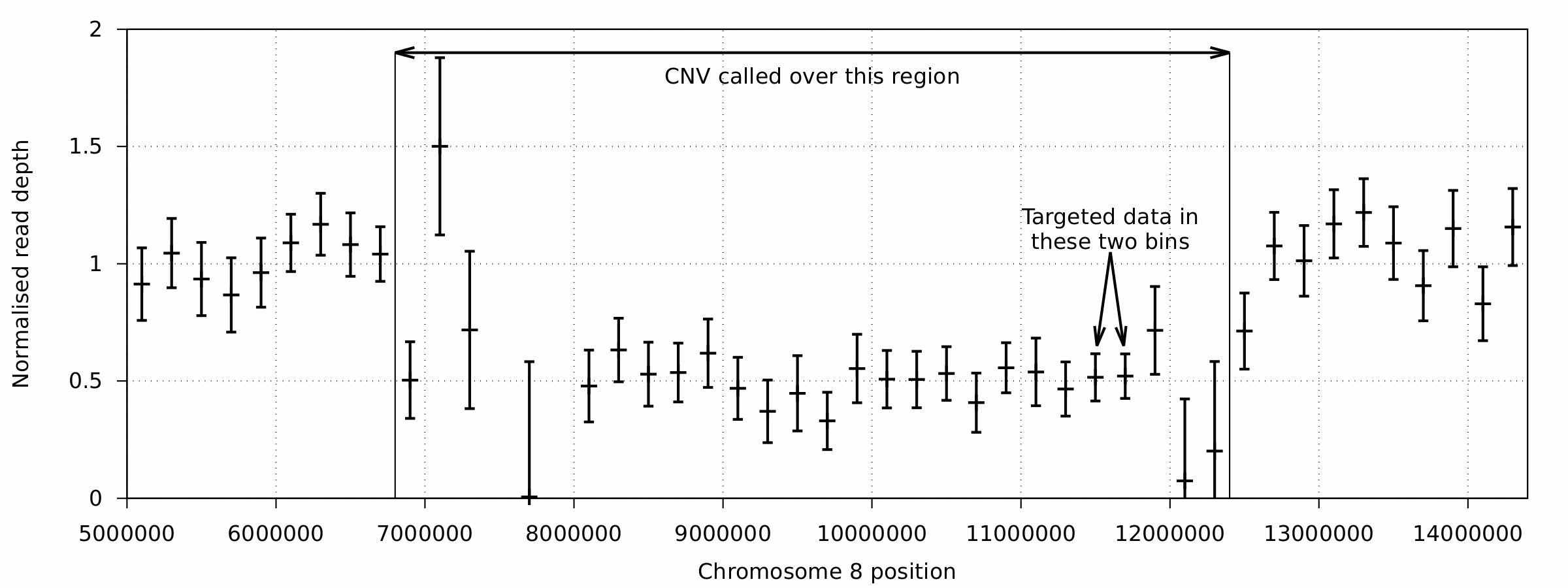


Fig A: An example of a single large CNV called by SavvyCNV in chromosome 8. The CNV is a heterozygous deletion of 8:6,800,000-12,400,000, which is a 5.6Mbp deletion. The CNV analysis uses a bin size of 200kbp, so the CNV covers 28 bins. The normalised read depth of each bin is shown as an error bar, where the error is the estimated error calculated by SavvyCNV. Bins either side of the deletion have a normal normalised read depth near 1, whereas bins inside the deletion have a normalised read depth near 0.5. This CNV overlaps a targeted gene in the sequencing capture, and this targeted data covers two separate bins, which are marked. Note that the error estimate for these two bins is slightly smaller than the other bins, partly because of the increased read count contributed by the targeted region of the capture, however the difference is small – the majority of evidence for this CNV comes from off-target data. Some of the bins have a large estimated error – this is caused by large tandem repeat regions which mean the read depth is highly variable between samples.


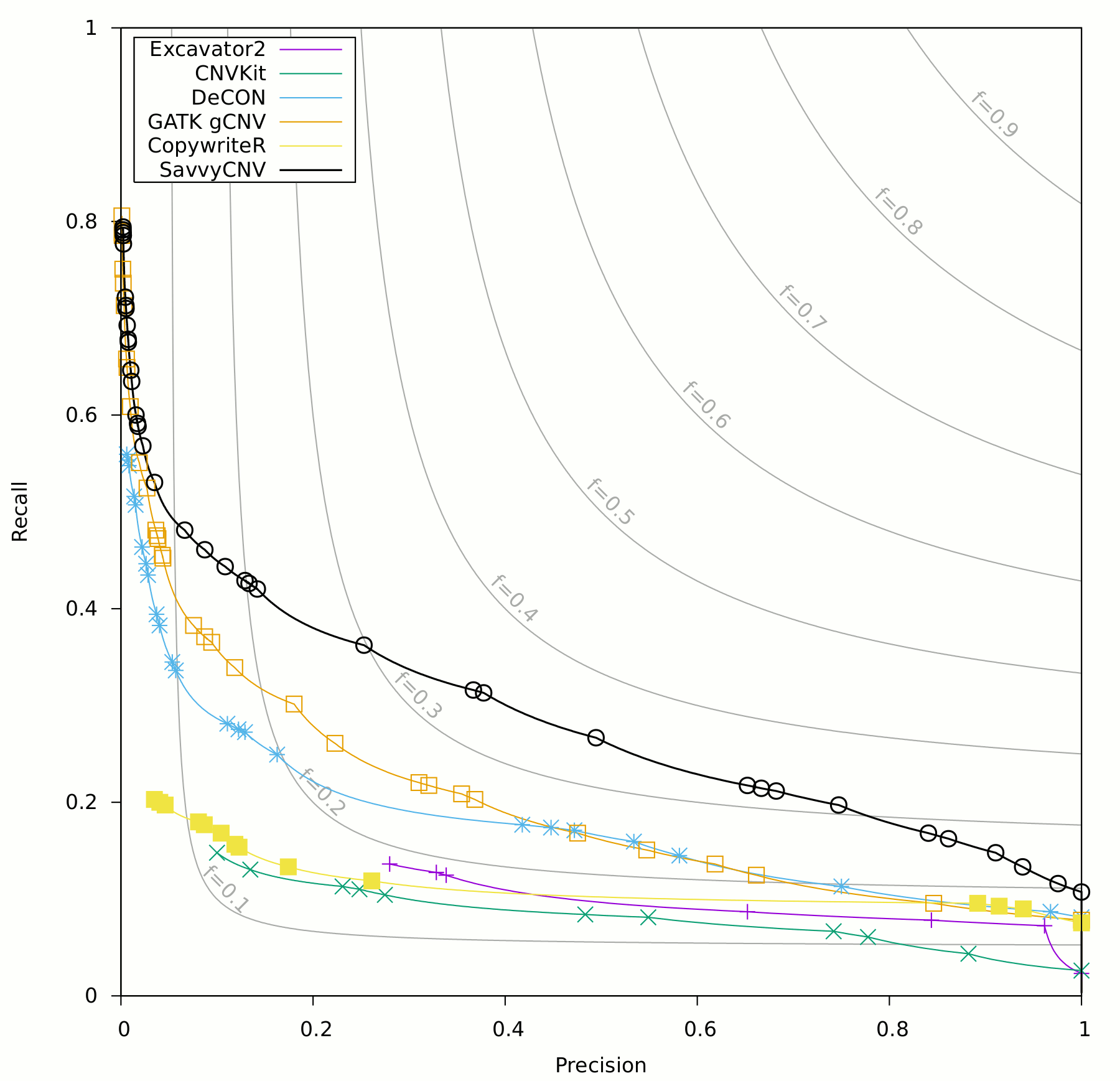
Fig B: Benchmarking off-target CNV calling from targeted panel data. This Fig shows the equivalent of Fig 1, but using a truth set called using Canvas on whole genome sequenced samples. The truth set used in Fig 1 was produced by analysing whole genome samples with GenomeStrip2 and filtered using variant allele fraction data. The data points on the plot are generated by a parameter sweep for each tool and show the precision and recall that can be achieved with each tool. The f statistic is the harmonic mean of precision and recall (see Materials and Methods for details).


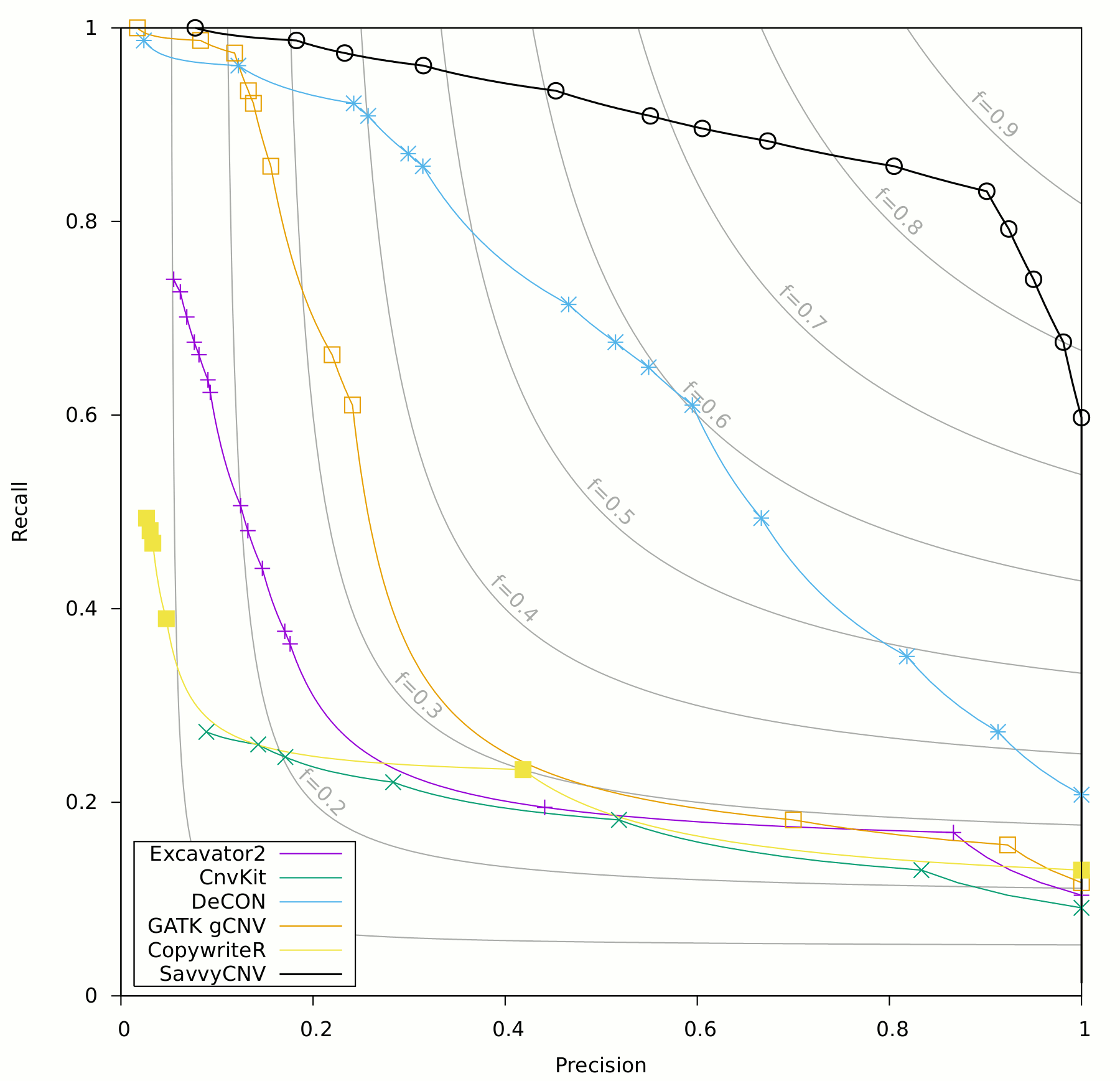
Fig C: Benchmarking off-target CNV calling from exome data. This Fig shows the equivalent of Fig 3, but using a truth set called using Canvas on whole genome sequenced samples. The truth set used in Fig 3 was produced by analysing whole genome samples with GenomeStrip2 and filtered using variant allele fraction data. The data points on the plot are generated by a parameter sweep for each tool and show the precision and recall that can be achieved with each tool. The f statistic is the harmonic mean of precision and recall (see Materials and Methods for details).

**Supplementary Methods – further information on how SavvyCNV calls CNVs**

SavvyCNV uses three strategies to improve the accuracy of its CNV calls. These are noise reduction using singular vector decomposition, the modelling of read depth error, and assuming that CNVs are non-mosaic. To determine whether these strategies actually do improve CNV call accuracy, we tested SavvyCNV using all three data sets, with different strategies instead. Fig D in S1 Text shows the effect of switching off singular vector decomposition. Fig E in S1 Text shows the effect of using different read depth error modelling. Fig F in S1 Text shows the accuracy of the error modelling strategies investigated. Fig G in S1 Text shows the effect of allowing the detection of mosaic CNVs. Fig H in S1 Text shows how the CNV detection ability of SavvyCNV depends on the number of samples that are analysed in a single batch.

Fig D: Shows the improvement in precision/recall due to the error correction strategy used by SavvyCNV, for all three data sets. By default, SavvyCNV uses singular vector decomposition (SVD), which identifies biases common to multiple samples, which can be caused by differences in sample handling or chemistry. The "normal" line shows the default configuration, while the "No SVD" line is with SVD switched off.
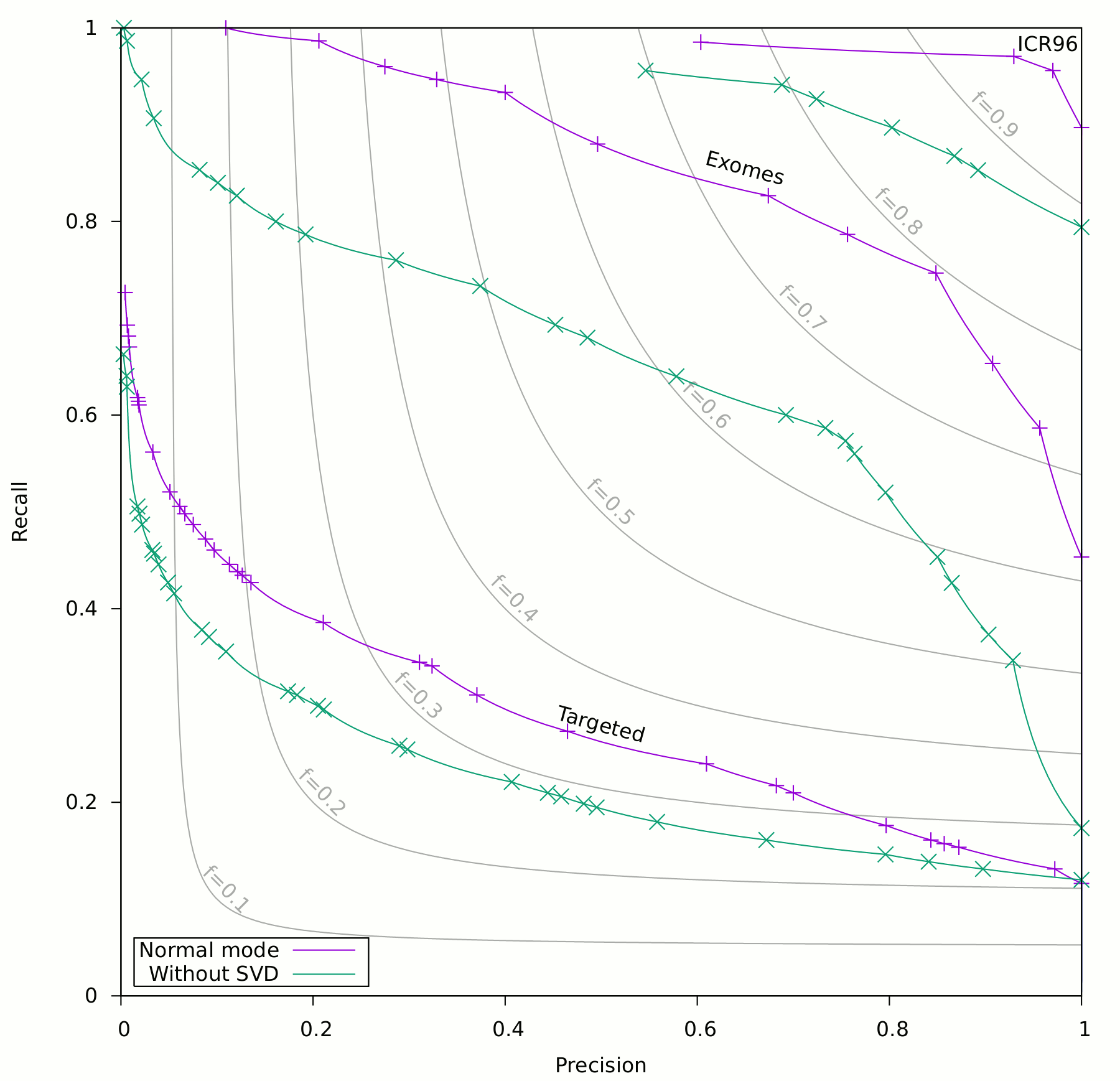


Fig E: Shows the improvement in precision/recall due to the error modelling strategy used by SavvyCNV. By default, SavvyCNV estimates the error in normalised read depth in each genomic location for a sample by calculating the standard deviation for that location across all samples, then scaling by the standard deviation for that sample across all locations. An alternative is to add the two error values. Other software assumes that the error is Poisson in nature, and can therefore be calculated from the read depth. The error estimate is used to determine whether the read depth in a genomic location is significantly outside the range expected for normal copy number. In reality, the actual error is larger than the Poisson method in some genomic locations. Modelling the error allows SavvyCNV to avoid making false CNV calls in these highly variable regions
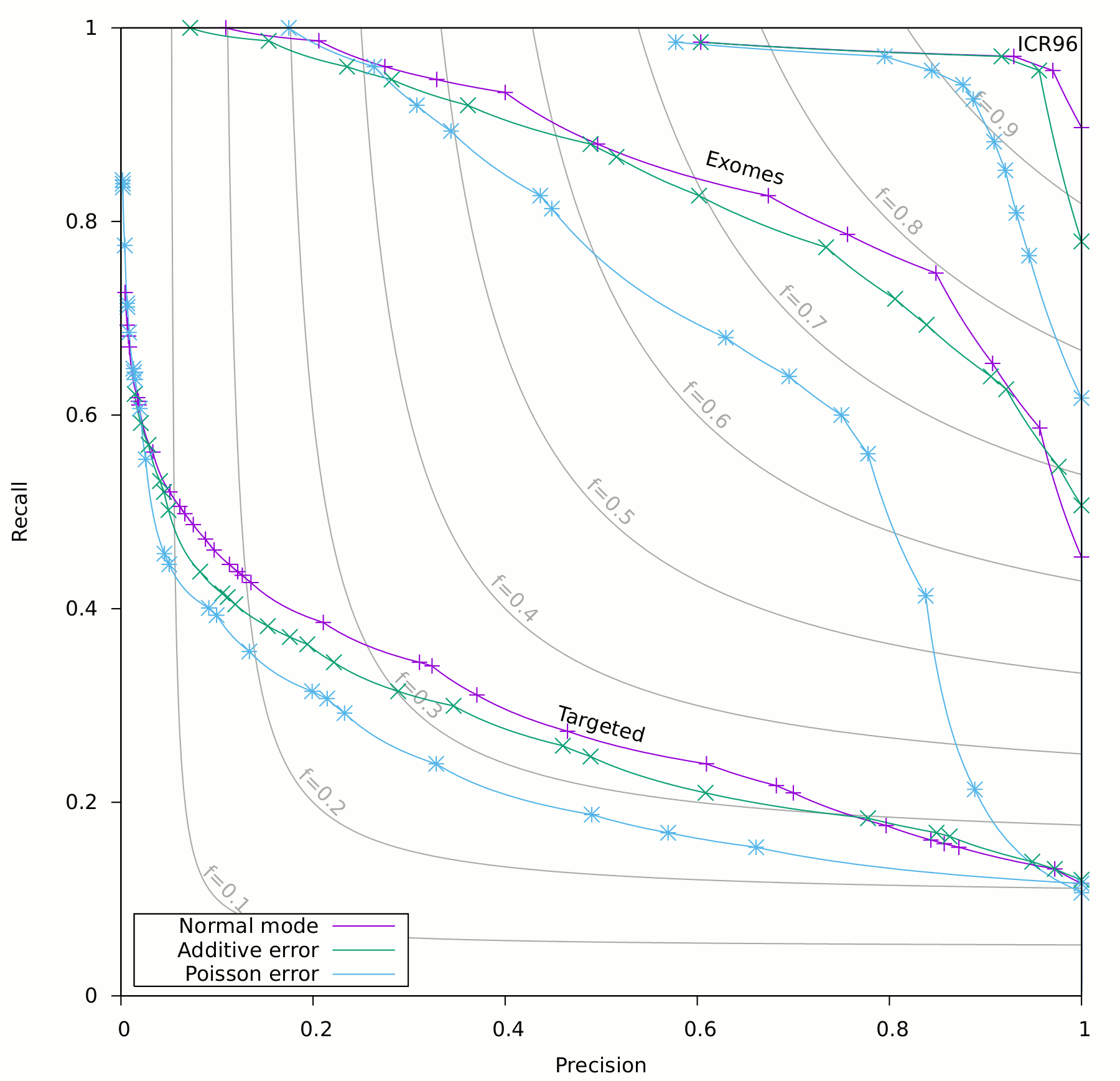


Fig F: Shows the error estimation accuracy of three strategies investigated during the development of SavvyCNV. The CNV detection ability of these three strategies is shown in Fig S5. SavvyCNV was used to analyse the read depths of around a thousand targeted samples with a bin size of 200kbp. The estimated error was calculated using the three strategies and listed alongside the actual normalised read depth value for each bin in each sample, giving a total of 32 million data points.
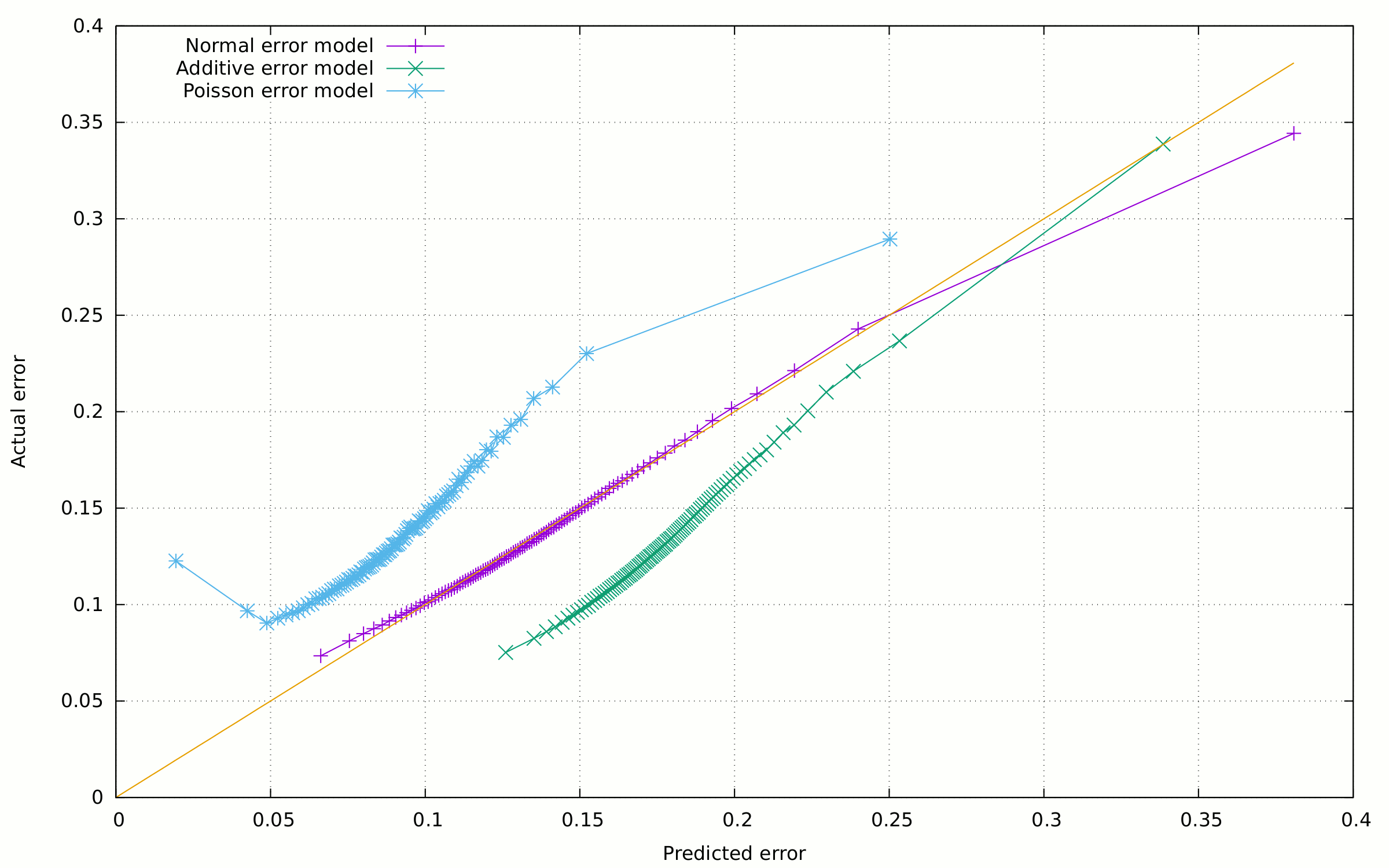
 These data points were then grouped into 100 sets with similar estimated error, and the standard deviation of the normalised read depth was calculated - this is the actual error. The Poisson error model calculates the estimated error by using the number of reads in the analysis bin, and it usually underestimates the actual error, however it does represent a theoretical minimum random error that the normalised read depth could have. The additive error is formed by adding the standard deviation of the genomic location across all samples with the standard deviation of the sample across all genomic locations. This usually overestimates the error, but this cannot be corrected by scaling. The normal error model multiplies the two error estimates together and divides by the mean error of all samples, and is highly effective at estimating the error in the normalised read depth. As a check, if this calculation yields an error lower than the Poisson error, then the Poisson error is used instead.


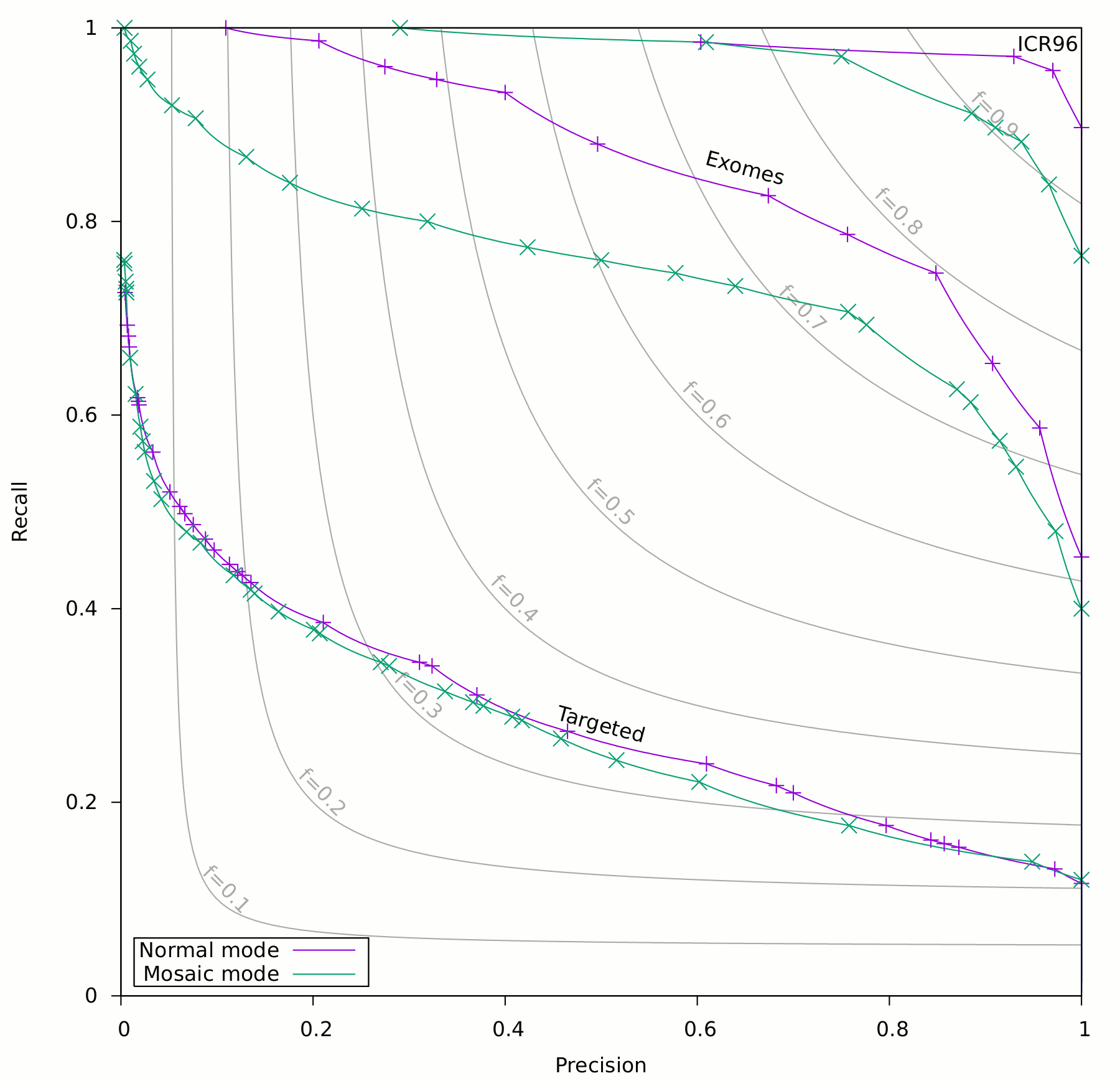
Fig G: Shows the improvement in precision/recall due to the non-mosaic assumption used by SavvyCNV. By default, SavvyCNV assumes that CNVs are not mosaic, although this is a configurable setting. This allows it to reduce the number of false positive CNV calls. In mosaic mode (and DeCON, Excavator2, and CNVKit), a CNV call is produced when the normalised read depth is significantly away from 1.0. In SavvyCNV's default mode (and GATK gCNV), the read depth must also be closer to 0.5 or 1.5 (representing a whole heterozygous deletion or duplication) than 1.0 in order for a CNV to be called.


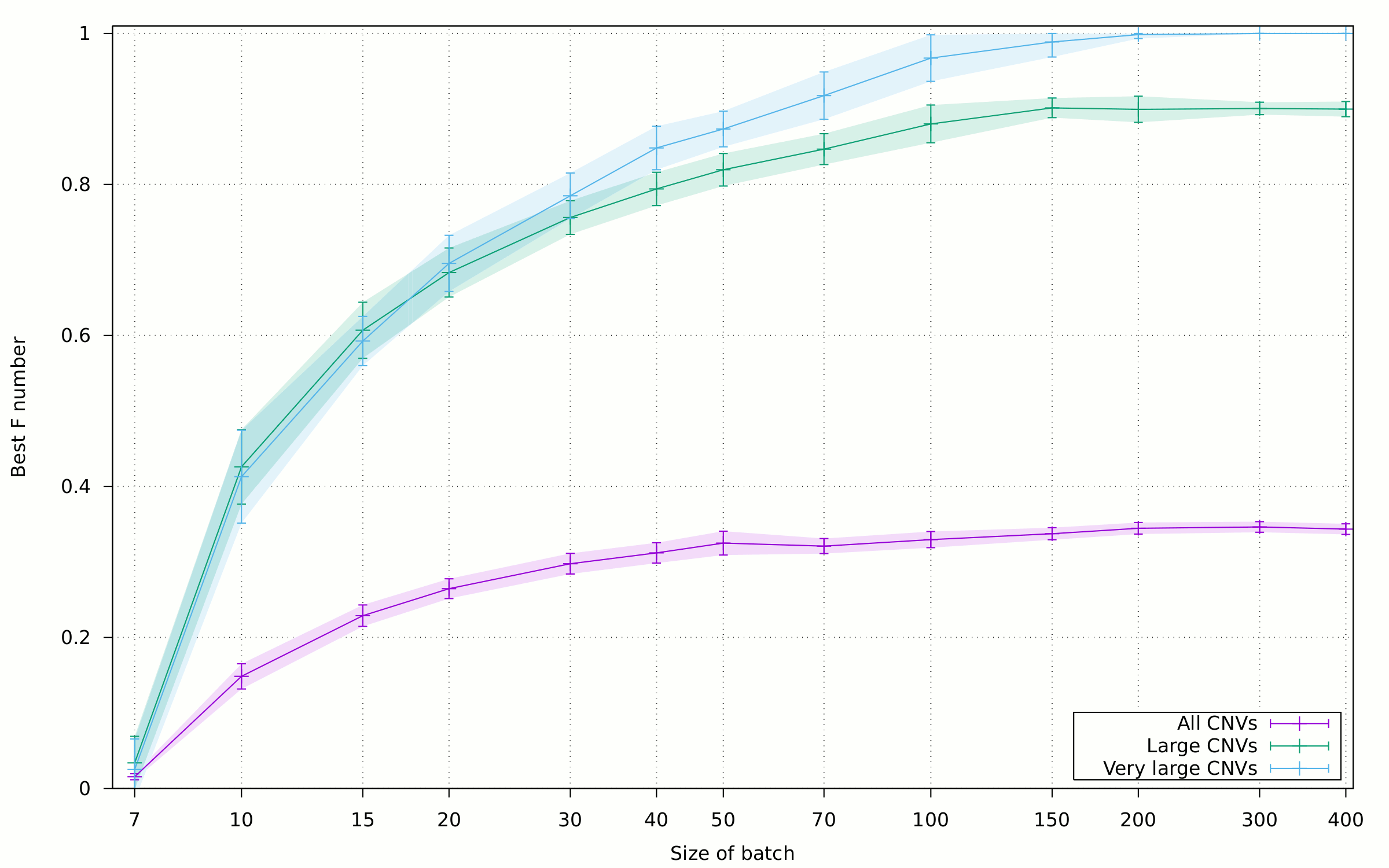
Fig H: Shows how the CNV detection ability of SavvyCNV depends on the number of samples that are analysed in a single batch. SavvyCNV detects regions where the read depth of a sample is higher or lower than expected, and so it must have other samples to compare the test sample to. A larger number of available samples improves the ability of SavvyCNV to correct for noise and determine the expected read depth for each sample, to better detect deviations from that expected read depth. We divided our set of targeted sequencing samples into randomly assigned batches sized between 7 and 400 samples, and ran SavvyCNV with multiple configuration settings as described for the main experiment that produced Fig 1 for each group size. The maximum f statistic was calculated for each group size. This process was repeated 25 times with randomly-assigned groups. This shows that the detection power of SavvyCNV for this set of samples is lower when fewer samples are analysed. The power to detect all CNVs is fully reached when there are at least 50 samples analysed. The power to detect larger CNVs continues to increase with larger sample batches up to a maximum with 200 in each batch.
