# Supplementary material for: SavvyCNV: Genome-wide CNV calling from off-target reads
Source: PLoS Comput Biol. 2022 Mar 16;18(3):e1009940. doi: 10.1371/journal.pcbi.1009940 (PMC8959187; doi:10.1371/journal.pcbi.1009940)
Supplement: Attachment — Submitted filename: SavvyCNV_reviewer_responses - final (1).docx [file pcbi.1009940.s002.docx]

# SavvyCNV paper reviewer responses

**Correction to manuscript**

During re-analysis during the review process we discovered an error in the analysis of the CopywriteR software. Results for CopywriteR were better than we originally reported. We have amended our figures and tables with the correct results. SavvyCNV still performs better than CopywriteR on off-target and on-target calling for all sizes of CNV so our overall conclusions are not affected.

Reviewer #1: The paper by Laver et al presents SavvyCNV, a tool for CNV calling from hybrid capture sequencing data which can make use of off-targets reads. While plenty of tools have been developed for CNV calling from exome and panel sequencing data, CNV calling cannot be considered as a solved problem: concordance between calls from different tools is low, and false negatives and false positives are common. Hence there is still a high (and with the increasing use of panel and exome sequencing for diagnostics growing) need for improved CNV calling methods.

The authors benchmark SavvyCNV against five other tools in three settings: off-target calling from exome data, off-target calling from panel data, and on-target calling from panel data. For benchmarking calling using off-target reads, an in-house data set with panel sequencing data from 2591 patients and exome sequencing data from 86 patients. The truth set for these benchmarks was obtained from deep whole genome sequencing of 170 panel-sequenced and 42 exome-sequenced samples. As truth, the authors considered CNV calls obtained by GenomeStrip 2.0 from this genome sequencing data. On-target calling was benchmarked using the previously published IRC96 dataset. For all tools, parameter sweep was performed and performance was evaluated in precision recall curves. SavvyCNV had the best overall performance in all shown comparisons. In the supplement, the authors add results which demonstrate the impact of three approaches used in the CNV calling process of SavvyCNV.

Overall, the authors have performed a comprehensive comparison to demonstrate the superiority of their tool in the tested scenarios. Based on these results, SavvyCNV appears to be a considerable improvement compared to the other callers included in the benchmark and thus might be of high value for a broader community.

However, there are still some issues which should be addressed:

Major issues:

1. The truth set for off-target calling can be expected to contain false negatives and false positives. The statement of the authors, that these will affect all tools to the same amount is not necessarily true: tools with a similar error profile as GenomeStrip 2.0 will have an advantage in this benchmark and might even appear to have a better performance than a perfect caller. Although one could hypothesize that calling from whole genome data is sufficiently more powerful to make the errors of GenomeStrip negligible, an additional comparison using an independent truth set would make the manuscript stronger. The authors could for example use the recently compiled validation set on NA12878 published by Gordeeva et al. (Scientific Reports 2021).

####>We agree with the reviewer that the increased data from genome sequencing compared to off-target calling should make calling CNVs from such data sufficiently accurate to use as a truthset. However, we appreciate the point that it would be good to verify that our choice of GenomeStrip2 to call the truthset has not influenced the results. To address this, we have re-called the truthset using the widely used CNV caller – Canvas (Roller et al 2016). When our tool comparisons are repeated on this new version of the truthset the results are similar to our original results. We have included these results as supplementary graphs.

We appreciate the suggestion of using NA12878 as an alternative truthset. However, the primary purpose of SavvyCNV is to call large, rare CNVs from off-target reads. NA12878 only has one single rare deletion >100kbp (according to ftp://ftp-trace.ncbi.nlm.nih.gov/giab/ftp/technical/svclassify_Manuscript/Supplementary_Information/Personalis_1000_Genomes_deduplicated_deletions.bed).

2. The methods descriptions in the manuscript are incomplete. This affects both the savvyCNV algorithm and the comparison. For example, it is not described at all how savvyCNV performs calling. There are only rather general descriptions of noise handling and the error model, but no in-depth methods; obviously, SavvyCNV employs a hidden Markov Model (mentioned in the CNV tools comparison section), but there is no description how it is implemented. Similarly, more details should be given about the CNV calling benchmark. How exactly have CNVs been compared? Was a CNV call considered as correct if it was partially overlapping with a reference call but had shifted start and/or end coordinates? Was the classification just gain or loss, or was there a discrimination of hemi- and homozygous deletions and different levels of gains.

####> We have added more details on both the SavvyCNV algorithm and how the tool comparisons were conducted to the methods section of the paper. To answer your specific questions:

- Yes SavvyCNV employs a hidden Markov Model with three states.
- Yes a CNV call was considered correct if it overlapped the reference call and was classified as correctly “loss” or “gain” with no further distinction between hemi/hetero/homozygous variants.

3. More information should be included to help potential users to decide if savvyCNV could be the right tool for their application. In particular, it would be important to know how many samples are needed for an analysis and how similar these samples have to be. Many users won’t have access to such a big collection of samples as used here for exome and in particular panel off-target calling.

####> The reviewer makes an excellent suggestion. We have repeated the sensitivity/specificity experiment using smaller batches of samples in each SavvyCNV run to determine how the number of available control samples affects the accuracy of the CNV calls. Results are shown in supplementary figure S8.

Regarding how similar the samples have to be, it is hard to speculate. However we have seen multiple examples where relevant CNVs were detected when samples with different characteristics were mixed, although the accuracy is always improved when they are segregated. The largest danger when mixing samples is that the singular vector decomposition will identify the differences between the dissimilar samples as the “largest pattern”, and this pattern will be removed if it is among the first five singular vectors. This can allow accurate CNV calling to proceed ignoring the difference between the samples, but it also consumes a singular vector that would be better used to cancel out other sample variation, such as handling procedures and GC-bias. For example, a mixture of male and female samples will result in the first singular vector representing the difference in read depths over the X and Y chromosomes, which will be cancelled out. CNVs in the X and Y chromosomes will still be called. This becomes more dangerous if a single outlier sample is processed with a set of similar samples. In this case, the “largest pattern” can be the entirety of the read depth variation in that one sample, including all the CNVs, which will be removed, resulting in a complete loss of CNV detection. I have recently added checks to SavvyCNV that will warn if this occurs.

4. The code and in particular the exact parameters used for each tool in the benchmark should be provided.

####> We have uploaded the code used to run the benchmarking to <https://github.com/exeter-matthew-wakeling/SavvyCNV_benchmarking> and included the link to it in the methods.

Minor issues:

1. Page 11, discussing mosaic or random noise. I agree that it is very likely noise, but most likely not random.

####> . We agree with the reviewer and have amended the paper to simply state that false positives are likely due to noise.

2. CNVkit was primarily designed for somatic CNV calling, and the documentation mentions that when used for germline calling it can be expected to show limited performance. This should be briefly added to the discussion.

####> We appreciate the suggestion and have included it in the discussion.

3. It seems that SavvyCNV has been developed and evaluated for germline CNV calling and not for somatic CNV calling. This should be mentioned in the manuscript; it could even be added to the title.

####> This is a good point. We have added germline CNV calling to appropriate places in the abstract, introduction and discussion to make clear this is the purpose of the tool.

Reviewer #2: # Overview

Detection of CNVs from targeted sequencing and exome data is an important problem in clinical genetics and there have been a wide range of tools published over the last decade. As noted by the authors however, most of these tools achieve mediocre precision when applied at acceptable sensitivity for clinical use, and further they typically can only operate well over the specific genes targeted by the specific assay, making it impossible to characterise the real span of larger genomic events even when they are detected.

SavvyCNV offers an exciting addition to this ecosystem of tools because it enables detection of larger events completely outside targeted regions and characterisation of the size and breakpoints of events that would otherwise be detected only within targeted regions. This is a valuable contribution and even while other tools do exist, SavvyCNV bring several novel additions to the process along with a high quality, well performing implementation.

In addition to reviewing the manuscript, I evaluated SavvyCNV on my own data. Although it required several iterations of tuning the parameters of the software, after doing that I was impressed with the speed and accuracy. It detected all known multi-exon CNVs and nearly all or all single-exon CNVs, which depended on the configuration parameters I chose. The software was easy to download, compile and run : it was refreshing to see a pure Java implementation with minimal dependencies, making it highly portable and performant.

Despite my good impressions of the software, I think the manuscript itself needs some additions before it is fit for scientific publication. The problems include insufficient description of methods, lack of clarity around terms used, missing essential details for reproducibility and failure to acknowledge significant weaknesses and limitations.

I have added specific notes below about areas that I think require addressing.

\# General Comments

- Overall it is a bit confusing that evaluation of off-target calling is combined with with on-target calling in various respects.

####> While the primary selling point of SavvyCNV is its ability to call off-target CNVs we were also keen to demonstrate that it could be used to call on-target CNVs and that a separate caller was not needed for this.

- When referring to "off target" CNVs, does this imply that there are no target regions overlapping the true CNV? Or only that off-target evidence is the primary signal for detection?

####> Off-target evidence is the primary signal, However, SavvyCNV will also use data from on-target regions. SavvyCNV is able to detect CNVs entirely from off-target signal.

We analysed the 68 CNVs found by SavvyCNV in the off-target analysis of targeted sequencing in the configuration shown in supplementary table S1. 17 of these CNVs overlap targeted regions. The majority of CNVs detected do not overlap targeted regions. We have added this result to the manuscript.

- If on-target regions are included, are the read counts included in SavvyCNV's algorithm? How does this affect the statistics when some bins contain spikes of very high coverage while others only contain low level coverage? It seems to me this would heavily distort, eg: the noise estimation which utilises standard deviation averaged across all the bins.

####> On-target regions are included in SavvyCNV’s analysis. The read counts are normalised before the noise level for a sample (standard deviation across all bins) is calculated. This means that bins that cover targeted regions do not disproportionately influence the noise calculation – each bin contributes equally to the noise.

I note that the software does not appear to take the target regions of the genome as a parameter; therefore it seems that in regular use both targeted regions and non-targeted regions would be considered by the tool together.

This leads to a contradiction: the primary claim of the manuscript is that SavvyCNV utilises off-target reads, but in reality it appears that most of its detections would be aided by at least *some* on target reads. To substantiate that off-target reads are really being utilised think there needs to be more detail given about (a) whether the results shown actually overlap on-target reads or not and (b) whether the off-target reads aided in characterisation of the true size / location of the CNV.

Further explanation around all of these issues is required.

####> We agree with the reviewer that sometimes on-target information is used to assist in the calling of CNVs. However, the targeted panel used in this study only covers 275kb (0.01% of the genome). As explained above there are multiple examples where CNVs were detected that do not overlap with any targeted regions. Regarding point (b), you are correct that off-target data aids in determining the true size of some CNVs. There are two examples within the ICR96 data set where CNVs were found by off-target calling to actually be large CNVs (15Mb and 56Mb) extending well beyond the targeted exons.

\# Specific Comments

> CNV calling can be optimised for precision or recall by adjusting configuration The section describes that SavvyCNV can be optimised for different requirements, however it does not present any details on how this is achieved. A summary of which parameters can be tuned and some idea of methodology of approach for doing so should be included. Is it possible without a dedicated truth set for the data under consideration? since each different type of data needs potentially different parameter values, it is not clear how a user can go about tuning these parameters in practice.

####> In that section we had tried to illustrate the different scenarios where you might want to choose precision over recall or vice-versa. However, we agree that it would be more useful if we linked to specific recommendations of which parameters can be tuned. We have now added this and referred users to the github page for precise details.

\## Results

> How much off-target read data is there?

How well SavvyCNV performs is critically dependent on how many off target reads there are. However this is a highly variable aspect of sequencing and capture methods. The manuscript should ideally give a bit more background explanation that off-target reads are influenced by the chemistry and laboratory process and may vary significantly between different contexts - this to some extent is an important limitation of the method and that should be acknowledged. It would be ideal to give a table showing a variety of exome captures and targeted panels with different chemistries and number of genes. Since this parameter is critical to operation of SavvyCNV, it would seem useful if there is some kind of QC statistic that is produced as part of an analysis that can inform the user of the abundance of off target reads that were observed.

####> This is an excellent point. We have added it to the discussion. We have published a tool on our github (CoverageOffTarget) to calculate the number of off-target reads in a set of samples and have also highlighted its availability in the discussion. This tool will also provide a suggestion of the bin size to use in SavvyCNV when analysing the samples.

> SavvyCNV can detect clinically relevant CNVs

In Table 1, presumably in some of these cases the span of the CNVs detected include some of the target regions of the panel (for example, the whole chrX aneuploidy). In these cases it should be clarified to what extent the off-target detection was aided by on-target reads. Presumably, SavvyCNV has enabled characterisation of the size of these events well beyond what would possible from the target regions alone. However, to substantiate this, it would be good for the results to include details showing how accurately SavvyCNV characterised the size / location of the CNV compared to orthogonal validation data for the events.

####>It is true that some CNVs detected overlap with targeted regions, and the extra read depth provided by the targeting undoubtedly helps with the detection. To show how much help this is, we have analysed an example of a CNV called in the data set, and shown the result in supplementary figure S1. The targeted region affects two of the 28 bins that form the CNV detection. Those two bins have a slightly lower estimated error than the rest due to this extra sequence data; however the difference is small.

In addition, we analysed the accuracy of the CNV boundary locations as called by all six of the CNV callers, for the off-target tNGS data set. Unfortunately, it is not possible to directly compare the CNV callers, because they find different sets of CNVs. However, SavvyCNV, GATK gCNV, and DeCON all perform very similarly, with a mean absolute error in boundary location varying proportionally with the bin size of the analysis. For a bin size of 200kbp, the error was around 90kbp for all three tools. The theoretical best accuracy should be a quarter of the bin size, so these three tools are not far from optimum. Excavator2, CopywriteR, and CnvKit had much higher location errors of 200kbp, 400kbp, and 600kbp respectively, although they detected too few CNVs to make this analysis sound.

Further, we would put forward the argument that since SavvyCNV is capable of detecting small CNVs using only off-target data, it should by inference be capable of detecting the off-target parts of larger CNVs, to correctly identify where the CNV boundaries are.

\# Assessment of Off-Target CNV Calling

The truth set was constructed using GenomeSTRiP. This seems a surprising choice given that there are many more well established whole genome sequencing CNV and SV callers now and GenomeSTRIP is designed for population scale data. A key issue with this approach is that GenomeSTRiP is also heavily read depth based, so it is susceptible to many of the same artefactual biases and could mean that many calls in the "truth" set are shared false positives between GenomeSTRiP and SavvyCNV. Similarly, many regions where GenomeSTRiP is unable to call CNVs will also apply to SavvyCNV, so use of GenomeSTRiP is questionable for characterising the false negative rate. While text acknowledges these issues in a minor way, this is somewhat buried and insufficiently highlighted (eg: in the conclusions).

####> As mentioned by reviewer 1, the increased data from genome sequencing compared to off-target calling should make calling CNVs from such data sufficiently accurate to use as a truthset. However, we appreciate the point that it would be good to verify that our choice of GenomeStrip2 to call the truthset has not influenced the results. To address this we have re-called the truthset using the widely used CNV caller – Canvas (Roller et al 2016). When our tool comparisons are repeated on this new version of the truthset the results are similar to our original results. We have included these results as supplementary graphs.

Furthermore, we appreciate that Canvas is still a read depth method. However, our method is for screening large CNVs. CNV detection methods from WGS data can be classified into boundary methods (read pair, clipped read, split read) and filled-in methods (read depth, variant allele fraction). The amount of boundary evidence does not vary with the size of the CNV, but the amount of filled-in data does, and so detection of large CNVs is best performed using read depth and variant allele fraction data. Our first truth set was generated from read depth data by GenomeStrip2 and validated using variant allele fraction data. For the sensitivity analysis the truth set was generated from read depth and variant allele fraction data by Canvas. To further improve on large CNV calling, we would have to obtain additional sequence data such as long read or optical alignment. We accept that the WGS data used to create the truth set and the tNGS data under test will both be biased against low complexity regions of the genome, and we have added to the discussion to make clear that this is a limitation.

\# Assessment of On-Target CNV Calling

- The manuscript doesn't state how much overlap was required for a CNV to be considered as successfully detected. I think this is quite important in the case of SavvyCNV, as it primarily detects larger events from off-target reads, but if these are only partially identified or identified in a fragmented way then it would affect the usability in many settings.

####> A CNV was deemed to have been detected if there was any overlap between the call made by the CNV caller and the truthset - this has now been added to the methods. In our tool comparisons fragmentation was effectively penalised by the fact that each non-overlapping call counted as a false call.

\# Materials and Methods

> Overview of how SavvyCNV calls CNVs

The overview is extremely brief and leaves many critical details out. For example, it is clear from the source code and alluded to in other parts of the manuscript that SavvyCNV applies a hidden markov model style segmentation algorithm using viterbi optimisation. However the overview does not include any mention of this part of the algorithm. Further, within the HMM the likelihood is evaluated by use of a normal distribution to model state probabilities, ie: reflecting a normal assumption for the distribution of read counts. These are commonly examined properties of CNV detection algorithms, and certain limitations arise from their characteristics (for example, utilising a normal model usually performs suboptimally for low read counts, and HMM based methods often have issues at the start / end points of the model because a neutral state is assumed as a starting point).

These details should be explained as important elements of the algorithm.

####> We have added further details on the algorithm to the methods. It is often the case that data analysis methods such as this that use multiple input values along with the error estimates to calculate a derivative output are highly dependent on the realism of the error estimates. Much of the failing of these methods can be traced to unrealistic error estimates. Three methods of modelling the error were tried while developing SavvyCNV. The simplest method uses just the number of reads in the bin and the Poisson distribution, which is essentially the method used by ExomeDepth/DeCON. This often underestimates the error, because the error is not just originating from Poisson noise. The chosen method takes the standard deviation of normalised read depths for a bin across all samples, and scales it by the standard deviation of normalised read depths for all bins in a sample, divided by the mean for all samples. If this error estimate is lower than the estimate provided by the Poisson distribution, then the Poisson estimate is used instead, which corrects for situations where the variation in read depth is unnaturally small, which could be caused by various reasons, including the absolute number of reads being very low. An alternative method adds the standard deviation of the bins across all samples with the standard deviation of all bins across one sample, but this tends to overestimate the error, and does not provide a good fit even when scaled to account for the fact that two measurements of error are added. We took all the read depth values for a SavvyCNV run, and grouped them by estimated error, then calculated the actual standard deviation in each group. The group estimated error is plotted against the actual calculated error in supplementary figure S6 for these three error estimation methods. It is clear that the chosen error estimation method provides a realistic estimate in all cases.

Using the Poisson distribution to adjust the error estimation should alleviate most of the trouble with the normal approximation when read counts are low. We accept that the normal approximation will still not be perfect in these cases.

We confirm that the HMM used by SavvyCNV starts and ends each chromosome with a normal copy number state. Therefore, a CNV detection must be supported by enough evidence to override the transition probability twice (once at each end of the CNV) regardless of whether the CNV is in the middle or the end of a chromosome.

> Dosage levels must cross the mid-point between 1 and 0.5 or 1.5 before they become evidence of a CNV.

This increases sensitivity

It doesn't seem possible that this could increase sensitivity because it acts purely to remove CNVs from the result set. Presumably the intent here is that it increases sensitivity at a given specificity?

####> The reviewer is correct and we have amended the text.

Further, this strategy seems likely to produce false negatives as even slightly imperfect normalisation can easily result in a CNV dosage estimate fractionally above 0.5, especially in the case of single-bin CNVs that would have only a point estimate of the dosage. Did the authors examine the strategy of using a statistical model for this element rather than a fixed threshold / cutoff?

####> The dosage needs to cross the mid-point between 1.0 and 0.5, which is 0.75 (approximately - the probability calculation method makes it slightly away from 0.75). The software does use a statistical model for this. The probability of the read depth for each bin being a deletion, duplication, or normal dosage is calculated using an approximation of the cumulative normal distribution, where the normal distribution has a standard deviation of the noise calculated for that bin. The mid-point between deletion and normal is where P(d<=0.5) = P(d>=1.0).

> CNV tool comparisons

The manuscript states that a range of parameter settings were used for each tool and the best chosen for each one. The parameters used for analysis with each tool are insufficiently described (either here, or where results are presented elsewhere). However I could not find either the ranges (or values) evaluated or the optimal values chosen for most of the callers (including SavvyCNV). For example, the bin size is stated as a user selectable parameter of SavvyCNV but the value chosen is not documented in the results, materials and methods or the supplementary information. These details are important to ensure reproducibility of the work. Similarly for the transition probability and number of SVD components - if these are left at their defaults then this could simply be stated.

####> The range of configuration values is included in the methods section, under the “CNV tool comparisons” subheading. We have uploaded the code used to run the parameter sweep / benchmarking to https://github.com/exeter-matthew-wakeling/SavvyCNV_benchmarking and included the link to it in the methods. One thing to note is that there is not one optimal configuration - there is an optimal configuration for each position on the precision-recall curve. We have added the configuration and filtering parameters that produced the relevant results to supplementary tables S1, S2, and S3. The transition probability was varied as described for SavvyCNV and DeCON. The number of SVD components zeroed was left at its default setting.

A particular problem seems to arise in the selection of bin size. I evaluated SavvyCNV on internal data sets that we have used to validate CNV detection for exome sequencing. It was apparent in this process that choosing different bin sizes could make deletions in our truth set appear and disappear from the results. This effect primarily seems to be driven by the luck of how much of a bin (or how many bins) end up overlapping the event of interest. If bins are too large, small events cannot be captured, while if they are too small, they become vulnerable to noise and weak power.

####> This is true. We have added a note to the discussion that if the aim of the user’s study is to detect large CNVs then a large bin size may be preferable for this reason. We have added a section to the SavvyCNV github documentation describing how to control sensitivity and specificity using the configuration options.

\# Software Suggestions

- The output file for SavvyCNV does not contain any headers. This makes it difficult to parse and

process in downstream tools (eg: Pandas, Excel, etc).

####> This is an excellent suggestion. We have added the option to include a header.

- The output file is referred to in the documentation as CSV format but is actually written as a tab

separated file

####> We thank the reviewer for spotting this mistake and have now corrected it in the documentation.

- The output file associates each call to the binned data file that the call came from. This required

me to add separate tracking outside of SavvyCNV to relate the files to each sample. Although it might

be normal to do this implicitly, eg: by naming the input files by sample, I feel it would be more

friendly if there was a way to identify the actual sample.

####> We agree with the reviewer and intend to fix this in the future.

Reviewer #1: No: Code and exact calls to tools used in the benchmark have not been made available. As specified in the data availability statement, also the in house dataset used in the benchmark could not be made available due to patient privacy issues.

####> We have uploaded the code used to run the benchmarking comparisons to https://github.com/exeter-matthew-wakeling/SavvyCNV_benchmarking and included this link in the methods.

Reviewer #2: No: The authors have explained that part of their validation data set is clinical data that cannot be made public for patient confidentiality reasons. I am comfortable with this personally but obviously journal policy should be applied according to your own discretion.
